# Classifying migraine subtypes and their characteristics by latent class analysis using data of a nation-wide population-based study

**DOI:** 10.1038/s41598-021-01107-7

**Published:** 2021-11-03

**Authors:** Wonwoo Lee, In Kyung Min, Kwang Ik Yang, Daeyoung Kim, Chang-Ho Yun, Min Kyung Chu

**Affiliations:** 1 Department of Neurology, Severance Hospital, Yonsei University College of Medicine, 50-1 Yonsei-ro, Seodaemun-gu, Seoul, 03722 Korea; 2 Biostatistics Collaboration Unit, Department of Biomedical Systems Informatics, Yonsei University College of Medicine, Seoul, Korea; 3 Department of Neurology, Soonchunhyang University College of Medicine, Cheonan Hospital, Cheonan, Korea; 4 Department of Neurology, Chungnam National University Hospital, Daejeon, Korea; 5 Department of Neurology, Bundang Clinical Neuroscience Institute, Seoul National University Bundang Hospital, Seongnam, Korea

**Keywords:** Headache, Migraine, Neurology

## Abstract

Migraine neither presents with a definitive single symptom nor has a distinct biomarker; thus, its diagnosis is based on combinations of typical symptoms. We aimed to identify natural subgroups of migraine based on symptoms listed in the diagnostic criteria of the third edition of the International Classification of Headache Disorders. Latent class analysis (LCA) was applied to the data of the Korean Sleep-Headache Study, a nationwide population-based survey. We selected a three-class model based on Akaike and Bayesian information criteria and characterized the three identified classes as “mild and low frequency,” “photophobia and phonophobia,” and “severe and high frequency.” In total, 52.0% (65/125) of the participants were classified as “mild and low frequency,” showing the highest frequency of mild headache intensity but the lowest overall headache frequency. Meanwhile, “photophobia and phonophobia” involved 33.6% (42/125) of the participants, who showed the highest frequency of photophobia and phonophobia. Finally, “severe and high frequency” included 14.4% (18/125) of the participants, and they presented the highest frequency of severe headache intensity and highest headache frequency. In conclusion, LCA is useful for analyzing the heterogeneity of migraine symptoms and identifying migraine subtypes. This approach may improve our understanding of the clinical characterization of migraine.

## Introduction

Migraine is a common condition that affects approximately 5–15% of the general population^1^. Despite its debilitating effects, there is currently no definitive biomarker or single symptom of migraine; thus, its diagnosis is based on self-reported symptoms. The third edition of the International Classification of Headache Disorders (ICHD-3) requires fulfilling at least two of four typical headache characteristics and at least one typical combination from four accompanying symptoms for a migraine diagnosis^2^. Therefore, individuals with migraine have varying symptom combinations.

Identifying the symptom subtypes of a disorder may help to better characterize typical and atypical populations, improving precision diagnosis and treatment^3^. In this context, several approaches have been used to identify the heterogeneity of migraine symptomatology^4–11^. Statistical methodologies, including factor analysis, cluster analysis, discriminant function analysis, factor mixture modeling, latent trait analysis and latent class analysis (LCA), have been used to identify the subtypes of clinical symptoms^8,10,12^. Among them, LCA is the most commonly used methodology to identify subgroups of migraine according to their symptoms and comorbidity^11,13^. It is a subset of structural equation modeling used to find subtypes of cases in multivariate categorical data and allows detection of the presence of latent classes (the disease entities), creating patterns of association in the symptoms^14^.

Previous studies on subtyping of migraine symptoms were mostly performed during genetic analyses among individuals with headache, mostly using data of twins and their relatives^4–9^. Although these subtyped migraine symptoms, involved individuals included both non-migraine headache and those with migraine. Further, although two studies have subtyped clinical symptoms exclusively in individuals with migraine, these studies used cohort data of health professionals^15,16^. Consequently, these studies may not properly reflect the clinical symptoms of those in the general population.

The Korean Sleep-Headache Survey (KSHS) was a nationwide population-based cross-sectional survey about headaches and sleep that provides an opportunity to evaluate and to subtype clinical symptoms of individuals with migraine in a general population. This study aimed to identify the subtypes of migraine symptoms in individuals with migraine through LCA using the data of KSHS.

## Results

### Participants

In total, 2501 individuals completed the KSHS in 2018. The distribution of sex, age and educational level was not significantly different from those in the overall Korean population (Supplementary Table S1). Overall, 1186 participants reported experiencing a headache during the previous year; of them, 125 participants (4.99%, 95% confidence interval [CI] 4.14–5.85%) were diagnosed with migraine. They were aged 45.8 ± 15.0 years and included 50 males and 75 females.

### Model selection and classification of classes

Three models were proposed and assessed as to their likelihood of prediction error. The Akaike information criterion (AIC) value was the lowest in the four-class model, while the Bayesian information criterion (BIC) value was the lowest in the two-class model (Table 1). Considering the discrepancy between the lowest AIC and BIC values within the models, we selected the three-class model because its BIC was lower than that of the four-class model and because it best described the different characteristics of the latent classes clinically. Both the standard errors of estimated prior probabilities of each class of the latent class membership and the class-conditional response probabilities of the indicators were within acceptable range (Supplementary Tables S2 and S3).

**Table 1:** Model selection criteria for latent class analysis.

Number of latent classes	2	3	4
AIC	1453	1448	1447
BIC	1524	1556	1592

*AIC* Akaike information criterion, *BIC* Bayesian information criterion.

### Demographic characteristics of the three classes

The model allowed sorting into Classes 1, 2 and 3, which identified 65, 42 and 18 individuals, respectively (Table 2). There were no significant differences in age, sex, residential area, education, income, body mass index and proportion of obesity among the three classes. The prevalence of comorbidities (hypertension, diabetes and dyslipidemia) was also similar.

**Table 2:** Sociodemographic, anthropometric and metabolic characteristics of participants with migraine.

	Total (n = 125)	Class 1 (n = 65)	Class 2 (n = 42)	Class 3 (n = 18)	P-value
**Age, years**	45.8 ± 15.0	45.8 ± 15.9	45.3 ± 13.6	46.8 ± 15.8	0.934
**Sex**					0.137
Male	50 (40.0%)	23 (35.9%)	16 (38.1%)	11 (61.1%)	
Female	75 (60.0%)	42 (64.6%)	26 (61.9%)	7 (38.9%)	
**Education**					0.223
Middle school or lower	16 (12.8%)	11 (16.9%)	5 (11.9%)	0 (0.0%)	
High school	52 (41.6%)	23 (35.4%)	18 (42.9%)	11 (61.1%)	
College or higher	57 (45.6%)	31 (47.7%)	19 (45.2%)	7 (38.9%)	
**BMI**, kg/m^**2**^	22.60 (21.22, 23.64)	22.63 (21.02, 24.08)	22.12 (21.28, 22.95)	22.76 (22.55, 24.99)	0.276
**Hypertension**	27 (21.6%)	14 (21.5%)	10 (23.8%)	3 (16.7%)	0.827
**Diabetes**	13 (10.4%)	7 (10.8%)	6 (14.3%)	0 (0.0%)	0.314
**Dyslipidemia**	11 (8.8%)	3 (4.6%)	7 (16.7%)	1 (5.6%)	0.081

Data are presented as the mean ± standard deviation, median (interquartile range), or n (%).

*BMI* body mass index.

Statistical method for comparison of the three groups: one-way analysis of variance for normally distributed variables; Kruskal–Wallis test for non-normally distributed variables; Chi-square or Fisher’s exact test for categorical variables.

### Headache characteristics under the ICHD-3 migraine diagnostic criteria

The headache characteristics according to the ICHD-3 diagnostic criteria of migraine among the three classes are summarized in Table 3. The results of multiple comparisons are shown in Supplementary Table S4. There were significant differences in the distribution of headache intensity, unilateral pain, aggravation by routine physical activity, nausea, vomiting, photophobia and phonophobia among the three classes but not in pulsating quality. Unilateral pain and mild headache intensity were the most frequent symptoms, and aggravation by routine physical activity was the least frequent in Class 1, while photophobia and phonophobia were the most frequent in Class 2. Class 3 showed the highest frequency of nausea, aggravation of routine physical activity and severe headache intensity.

**Table 3:** Characteristics of headache under the diagnostic criteria of migraine in the third edition of the International Classification of Headache Disorders.

	Total (n = 125)	Class 1 (n = 65)	Class 2 (n = 42)	Class 3 (n = 18)	P-value
**Headache intensity**					0.012^†c^
Mild	21 (16.8%)	13 (20.0%)	8 (19.1%)	0 (0.0%)	
Moderate	78 (62.4%)	35 (53.9%)	31 (73.8%)	12 (66.7%)	
Severe	26 (20.8%)	17 (26.2%)	3 (7.1%)	6 (33.3%)	
**Unilateral location**	87 (69.6%)	60 (92.3%)	19 (45.2%)	8 (44.4%)	< 0.001^†ab^
**Pulsating quality**	98 (78.4%)	48 (73.9%)	33 (78.6%)	17 (94.4%)	0.171
**Aggravation by routine physical activity**	59 (47.2%)	8 (12.3%)	33 (78.6%)	18 (100.0%)	< 0.001^†ab^
**Nausea**	112 (89.6%)	63 (96.9%)	31 (73.8%)	18 (100.0%)	< 0.001^†a^
**Vomiting**	56 (44.8%)	34 (52.3%)	20 (47.6%)	2 (11.1%)	0.007^†bc^
**Photophobia**	45 (36.0%)	1 (1.5%)	42 (100.0%)	2 (11.1%)	< 0.001^†ac^
**Phonophobia**	66 (52.8%)	22 (33.9%)	42 (100.0%)	2 (11.1%)	< 0.001^†ac^

Data are presented as n (%).

^†^Significant P-value.

^a^Significant difference between Class 1 and Class 2 in multiple comparisons.

^b^Significant difference between Class 1 and Class 3 in multiple comparisons.

^c^Significant difference between Class 2 and Class 3 in multiple comparisons.

The multiple comparisons revealed that the frequency of unilateral pain, aggravation by routine physical activity, nausea, photophobia and phonophobia was significantly different between Class 1 and Class 2. Meanwhile, the frequency of unilateral pain, aggravation by routine physical activity and vomiting differed significantly between Class 1 and Class 3. Headache intensity, photophobia and phonophobia were significantly different between Class 2 and Class 3.

### Clinical features of migraine outside the ICHD-3 diagnostic criteria

Headache frequency per month, headache duration (in minutes), osmophobia, impact of headache, visual aura, anxiety and fatigue were significantly different among the three classes (Table 4). Multiple comparisons showed that the frequency of osmophobia, visual aura, anxiety and fatigue was significantly different between Class 1 and Class 2 (Supplementary Table S4).

**Table 4:** Clinical features not included in the diagnostic criteria of migraine in the third edition of the International Classification of Headache Disorders.

	Total (n = 125)	Class 1 (n = 65)	Class 2 (n = 42)	Class 3 (n = 18)	P-value
**Monthly headache frequency**					
**n**	0.83 (0.33, 3.00)	0.50 (0.33, 2.00)	1.00 (0.42, 4.00)	1.50 (0.52, 7.00)	0.030^†^
**By category**					0.019^†b^
< 2	75 (60.0%)	44 (67.7%)	22 (52.4%)	9 (50.0%)	
≥ 2, < 8	33 (26.4%)	18 (27.7%)	11 (26.2%)	4 (22.2%)	
≥ 8, < 15	12 (9.6%)	1 (1.5%)	6 (14.3%)	5 (27.8%)	
≥ 15	5 (4.0%)	2 (3.1%)	3 (7.1%)	0 (0.0%)	
**Headache duration (min)**	300.0 (240.0, 480.0)	300.0 (240.0, 600.0)	300.0 (240.0, 465.0)	240.0 (240.0, 300.0)	0.030^†b^
**Osmophobia**	48 (38.4%)	13 (20.00%)	28 (66.67%)	7 (38.89%)	< 0.001^†a^
**Aura (VARS score ≥ 3)**	22 (17.6%)	6 (9.23%)	14 (33.33%)	2 (11.11%)	0.004^†a^
**HIT-6 score**	53.0 ± 8.9	51.3 ± 8.2	55.8 ± 10.0	52.3 ± 7.9	0.038^†a^
**MIDAS score**	1.00 (0.00, 3.00)	0.00 (0.00, 2.00)	1.00 (0.00, 4.00)	2.50 (0.25, 8.25)	0.069
**Anxiety (GAD-7 score ≥ 8)**	14 (11.2%)	3 (4.6%)	9 (21.4%)	2 (11.1%)	0.018^†a^
**Depression (PHQ-9 score ≥ 10)**	24 (19.2%)	9 (13.9%)	9 (21.4%)	6 (33.3%)	0.161
**Cutaneous allodynia (ASC-12 score ≥ 3)**	20 (16.0%)	10 (15.9%)	8 (19.1%)	2 (11.1%)	0.730
**Fatigue (FSS score ≥ 4)**	44 (35.2%)	16 (24.6%)	20 (47.6%)	8 (44.4%)	0.035^†a^
**FSS score**	3.67 (2.89, 4.33)	3.44 (2.78, 3.89)	3.89 (3.56, 4.44)	3.89 (3.00, 4.17)	0.021^†a^
**Excessive daytime sleepiness (ESS score ≥ 11)**	27 (21.8%)	14 (21.5%)	12 (29.8%)	1 (5.6%)	0.127
**ESS score**	8.00 (5.00, 10.00)	8.00 (5.00, 10.00)	8.00 (5.00, 11.00)	5.00 (4.25, 8.00)	0.167

Data are presented as the mean ± standard deviation, median (interquartile range), or n (%).

*VARS* Visual Analogue Rating Scale, *HIT-6* Headache Impact Test-6, *MIDAS* Migraine Disability Assessment, *GAD-7* General Anxiety Disorder-7, *PHQ-9* Patient Health Questionnaire-9, *ASC-12* The 12‐item Allodynia Symptom Checklist, *FSS* Fatigue Severity Scale, *ESS* Epworth Sleepiness Scale.

^†^Significant P-value.

^a^Significant difference between Class 1 and Class 2 in multiple comparisons.

^b^Significant difference between Class 1 and Class 3 in multiple comparisons.

^c^Significant difference between Class 2 and Class 3 in multiple comparisons.

### Characterization of the three classes

The three classes were named to reflect the unique characteristics of each class. Class 1 was characterized by mild and low frequency migraine and was the most common, with 52.0% (65/125) of the participants belonging to this classification. We named Class 1 as “mild and low frequency.” Class 2 as the next most common class was characterized by intermediate headache frequency and headache intensity. Nevertheless, this class showed the highest frequency of photophobia and phonophobia and was thus named “photophobia and phonophobia.” This class involved 33.6% (42/125) participants. Class 3 was the least common and was characterized by the highest headache intensity and headache frequency. We named this as “severe and high frequency.” This class included 14.4% (18/125) of the participants.

## Discussion

Migraine diagnosis is challenging because it does not present with a definitive single symptom or have a distinct biomarker. LCA in this study enabled us to classify each of the 125 participants with migraine into three classes. Each class showed distinct characteristics that can be briefly described as “mild and low frequency,” “photophobia and phonophobia” and “severe and high frequency”.

Several studies have attempted to subclassify migraine. Schürks et al. evaluated women with migraine and found three classes related to central nervous system (CNS) sensitization, attack frequency and pain location and aura and visual phenomena^15^. The “CNS sensitization” and “attack frequency and pain location” classes may correspond to our “severe and high frequency” and “mild and low frequency” classes, respectively. Both the “CNS sensitization” and “severe and high frequency” classes showed high headache frequency and aggravation by routine physical activity. Meanwhile, the “attack frequency and pain location” and “mild and low frequency” classes presented with frequent unilateral pain and mild headache intensity. However, photophobia and phonophobia were significantly related in our study, but not in Schürks et al.’s study. This discrepancy may be due to differences in study population, ethnicity, sex and analysis methods. Studies on twins and their relatives included individuals with migraine and non-migraine headache and selected a three- or four-class model. These studies found subtypes of high, intermediate, low and minimal frequency of migraine symptoms^4,5,7,8,13^.

Our study found several noteworthy findings on the subtypes and symptoms of migraine. First, Class 2 (photophobia and phonophobia) showed the highest rate of osmophobia (i.e., intolerance of or hypersensitivity to smells), which is a common symptom of migraine and has high specificity for migraine diagnosis^17,18^. Photophobia, phonophobia and osmophobia are sensory hypersensitivity symptoms^19^. Our findings support that there is a migraine subtype that presents with a high frequency of sensory hypersensitivity symptoms. Second, considering that the intensity and frequency of headache are key parameters for determining migraine severity^20^, we found that each symptom is closely related to severity. Unilateral pain was most common in Class 1 (mild and infrequent) and least common in Class 3 (severe and frequent). In contrast, aggravation by routine physical activity was the most frequent in Class 3 and the least frequent in Class 1.

Photophobia and severe headache intensity were prevalent in 36.0% and 20.8% of our participants, respectively. These rates are lower than those in Western countries but are similar to those in Asian countries. Approximately 68% to 84.5% of patients with migraine in Western countries have photophobia^21,22^, whereas the rates range from 53.9 to 67.4% in Asian countries^23,24^. Similarly, the prevalence of severe headache intensity is lower in Asian countries (19.5–38.0%) than in Western countries (60–85%)^23,25–27^.

LCA has been used in genetic research to capture underlying phenotypic and genetic variance in diseases with complex etiologies^7–9,13^. The three symptom subtypes of migraine identified in our study based on LCA may provide clues in understanding the pathophysiologic and genetic mechanisms of migraine. The presence of a subtype with a high frequency of photophobia and phonophobia suggests a shared, rather than an independent, pathophysiological mechanism in sensory hypersensitivity symptoms. In a similar context, the co-occurrence of a high headache frequency and severe headache intensity suggests that high headache frequency and severe headache intensity have overlapping pathophysiological mechanisms. In genetic studies, the identification of different subtype symptom profiles suggests the presence of different genetic loci in each phenotype.

This study had some limitations. First, the symptoms of migraine were evaluated based on the participant’s report. These symptoms can vary between attacks in the same person^28^. A headache diary could have been a more accurate method for symptom evaluation; however, it is difficult to use in epidemiological studies. Second, although we used data from a large-scale epidemiological study, the sample size of this study was relatively small for LCA. The minimum sample size for LCA is not fixed, although better performance is expected with larger sample sizes^29^. To overcome this issue, we used high-quality indicators with strong theoretical bases as variables, which allowed us to use a small sample and made classification and interpretation easy^30,31^. To avoid local maxima in the expectation–maximization algorithm, the latent class model was estimated 20 times using various initial parameter values; convergence failure was not present (Supplementary Tables S2 and S3).

However, the present study also had several strengths. First, our study used data from a nationwide population-based study that included a sample proportional to the population distribution of Korea. As such, the risk of selection bias was minimized. Second, we used a validated questionnaire for the diagnosis of migraine. Depression, anxiety, visual aura, fatigue, disability and impact of headache were also evaluated using validated Korean versions of the Patient Health Questionnaire-9 (PHQ-9), General Anxiety Disorder-7 (GAD-7), self-administered Visual Analogue Rating Scale (VARS), Fatigue Severity Scale (FSS), Migraine Disability Assessment (MIDAS) and Headache Impact Test-6 (HIT-6). This enabled an accurate evaluation of migraine and its clinical features. Third, we selected items of LCA based on the ICHD-3 diagnostic criteria of migraine, which comprised key characteristics of migraine. This allowed us to properly analyze and to compare findings. Other approaches of migraine subtyping including genetic, biochemical and neuroimaging studies are needed to verify our findings.

In this study, LCA identified three classes of migraine that showed distinct characteristics. These classes were “mild and low frequency,” “photophobia and phonophobia,” and “severe and high frequency,” in order of prevalence. LCA can be useful to analyze the heterogeneity of migraine symptoms and to identify migraine subtypes.

## Methods

### Survey

The method of sampling and survey of KSHS in 2018 has been previously described in detail^32^. Briefly, the survey used a two-stage clustered random sampling method proportional to population distribution of all Korean territories, except for Jeju-do, based on data from the 2017 population and housing census conducted by the National Statistical Office of Korea^33^. The target sample size was 2500 adults aged ≥ 19 years, and the estimated sampling error was ≤ 1.9%. The survey was conducted between October 2018 and November 2018 using face-to-face questionnaire interviews by trained interviewers. All interviewers were employees of Gallup Korea and had experiences in social survey. The questionnaire items included demographic characteristics, headache profiles, headache diagnosis, use of medical services, medical consultation, disability from headache, impact of headache, anxiety, depression and fatigue. This study was the secondary analysis of the KSHS.

### Diagnosis of migraine

Migraine was diagnosed based on the diagnostic criteria for migraine without aura in ICHD-3 (code 1.1)^2^. The diagnostic validity of the questionnaire has been previously reported^32^. Participants with a positive response to the question “Did you experience headache during the previous year?” and fulfilling the ICHD-3 criteria of migraine were diagnosed with migraine.

The presence of a visual aura was assessed using the self-administered VARS, for which the Korean version has been validated, with a score of ≥ 3 defined as having a visual aura^34,35^. Participants who fulfilled the ICHD-3 diagnostic criteria of migraine without aura but reported visual aura were diagnosed with migraine with aura (code 1.2)^2^.

### Assessment of physical and mental impact of headache

Migraine disability and the impact of headache were evaluated using the MIDAS and HIT-6 tools, respectively^36,37^. The Korean versions of the MIDAS and HIT-6 were previously validated^38,39^.

The level of anxiety was assessed using the GAD-7 tool^40^. Participants with a GAD-7 score of ≥ 8 were classified as having anxiety^41^. Depression was evaluated using the PHQ-9, with a PHQ-9 score of ≥ 10 defined as depression^42^. The Korean versions of the GAD-7 and PHQ-9 were previously validated^43,44^.

### Cutaneous allodynia, fatigue, and excessive daytime sleepiness

Cutaneous allodynia was evaluated using the Allodynia Symptom Checklist-12 (ASC-12), with an ASC-12 score of ≥ 3 being indicative of cutaneous allodyniac^45^. Fatigue was defined as an FSS score of ≥ 4^46^. Excessive daytime sleepiness was defined as an ESS score of ≥ 11^47^.

### Model selection of LCA

LCA was used to differentiate the natural subgroups of migraine. It is a type of finite mixture model, a statistical technique for the analysis of multivariate categorical data, used for examining relationships among observed variables. It stratifies the cross-classifications and probabilistic groups and characterizes them into unobserved (latent), unordered categorical variable classes^48,49^. In this way, observations with similar sets of responses on the manifest variables tend to cluster within the same latent classes^49^. In this study, LCA was performed using the “poLCA” package by R, which is a frequently used package for LCA^49^.

Given that all participants with migraine satisfied criteria A (total attack number), B (typical duration) and E (exclusion of other diagnosis), we used items in criteria C and D of migraine without aura (code 1.1)^2^ as variables for LCA. Headache intensity (mild, moderate and severe), unilateral location, pulsating quality, aggravation by routine physical activity, nausea, vomiting, photophobia and phonophobia were used as categorical variables in the gathered data. The headache frequency per month was categorized into a four-class variable (< 2, ≥ 2 but < 8, ≥ 8 but < 15 and ≥ 15). We evaluated the prior probabilities of latent class membership and class-conditional probabilities to check the quality of the indicators^30^. To avoid local maxima in the expectation–maximization algorithm, the latent class model was estimated 20 times using various initial parameter values.

The model with the best fit was selected primarily based on AIC and BIC, with their lowest values predicting the best fit model^48^. For comparison among classes, a one-way analysis of variance was used for normally distributed variables, while a Kruskal–Wallis test was used for non-normally distributed variables. A chi-square or Fisher’s exact test was used to compare categorical variables, as appropriate. Among observed variables in LCA and those with significant P values, normally distributed variables were compared using an independent t-test, whereas Dunn’s procedure was used for non-normally distributed variables^50^. Multiple comparison was conducted with the Bonferroni method, with P values represented as adjusted P values. All statistical analyses were performed using R version 3.6.0 (R Core Team, 2019)^51^. A two-sided P*-*value of < 0.05 was considered significant. There was no missing data except for monthly headache frequency in one participant with migraine which was adjusted as mean value.

### Ethical approval

This study was approved by the Institutional Review Board of Severance Hospital, Yonsei University (Approval No. 2018-1269-001). Written informed consent was obtained from all participants before the survey. Prior to obtaining written informed consent, all participants were given an explanation on the objective of the study and the data to be collected by interviewers. All procedures involving human participants were in accordance with the ethical standards of the institutional and/or national research committee as well as the tenets of the 1964 Declaration of Helsinki and its later amendments, or comparable ethical standards.

## Supplementary Information

Supplementary Information.

## Data Availability

The data used in this study are available from the corresponding author on reasonable request.

## References

[1] Stovner LJ (2018). Global, regional, and national burden of migraine and tension-type headache, 1990–2016: A systematic analysis for the Global Burden of Disease Study 2016. Lancet Neurol..

[2] Headache Classification Committee of the International Headache Society (IHS) (2018). The international classification of headache disorders, 3rd edition. Cephalalgia.

[3] Feczko E (2019). The heterogeneity problem: Approaches to identify psychiatric subtypes. Trends Cogn. Sci..

[4] Ligthart L (2008). A genome-wide linkage scan provides evidence for both new and previously reported loci influencing common migraine. Am. J. Med. Genet. B.

[5] Ligthart L (2010). Migraine symptomatology and major depressive disorder. Cephalalgia.

[6] Anttila V (2008). Consistently replicating locus linked to migraine on 10q22-q23. Am. J. Hum. Genet..

[7] Chen CC, Mengersen KL, Keith JM, Martin NG, Nyholt DR (2009). Linkage and heritability analysis of migraine symptom groupings: A comparison of three different clustering methods on twin data. Hum. Genet..

[8] Chen CC, Keith JM, Nyholt DR, Martin NG, Mengersen KL (2009). Bayesian latent trait modeling of migraine symptom data. Hum. Genet..

[9] Nyholt DR (2005). Genomewide significant linkage to migrainous headache on chromosome 5q21. Am. J. Hum. Genet..

[10] Lipton RB (2014). Improving the classification of migraine subtypes: An empirical approach based on factor mixture models in the American Migraine Prevalence and Prevention (AMPP) Study. Headache.

[11] Lipton RB (2018). Identifying natural subgroups of migraine based on comorbidity and concomitant condition profiles: Results of the chronic migraine epidemiology and outcomes (CaMEO) study. Headache.

[12] Kendler KS (1996). The identification and validation of distinct depressive syndromes in a population-based sample of female twins. Arch. Gen. Psychiatry.

[13] Nyholt DR (2004). Latent class and genetic analysis does not support migraine with aura and migraine without aura as separate entities. Genet. Epidemiol..

[14] Lazarsfeld PF, Henry NW (1968). Latent Structure Analysis.

[15] Schurks M, Buring JE, Kurth T (2011). Migraine features, associated symptoms and triggers: A principal component analysis in the Women's Health Study. Cephalalgia.

[16] Kossowsky J (2020). Association of genetic variants with migraine subclassified by clinical symptoms in adult females. Front. Neurol..

[17] Kelman L (2004). The place of osmophobia and taste abnormalities in migraine classification: A tertiary care study of 1237 patients. Cephalalgia.

[18] Wang YF, Fuh JL, Chen SP, Wu JC, Wang SJ (2012). Clinical correlates and diagnostic utility of osmophobia in migraine. Cephalalgia.

[19] Demarquay G, Mauguiere F (2016). Central nervous system underpinnings of sensory hypersensitivity in migraine: Insights from neuroimaging and electrophysiological studies. Headache.

[20] Sauro KM (2010). HIT-6 and MIDAS as measures of headache disability in a headache referral population. Headache.

[21] Stewart WF, Lipton RB, Liberman J (1996). Variation in migraine prevalence by race. Neurology.

[22] Russell MB, Kristiansen HA, Saltyte-Benth J, Kvaerner KJ (2008). A cross-sectional population-based survey of migraine and headache in 21,177 Norwegians: The Akershus sleep apnea project. J. Headache Pain.

[23] Wang SJ, Fuh JL, Young YH, Lu SR, Shia BC (2000). Prevalence of migraine in Taipei, Taiwan: A population-based survey. Cephalalgia.

[24] Takeshima T (2004). Population-based door-to-door survey of migraine in Japan: The Daisen study. Headache.

[25] Rasmussen BK, Jensen R, Schroll M, Olesen J (1991). Epidemiology of headache in a general population: A prevalence study. J. Clin. Epidemiol..

[26] Roh JK, Kim JS, Ahn YO (1998). Epidemiologic and clinical characteristics of migraine and tension-type headache in Korea. Headache.

[27] Radtke A, Neuhauser H (2009). Prevalence and burden of headache and migraine in Germany. Headache.

[28] Park JW, Chu MK, Kim JM, Park SG, Cho SJ (2016). Analysis of trigger factors in episodic migraineurs using a smartphone headache diary applications. PLoS ONE.

[29] Nylund-Gibson K, Ten Choi AY (2018). frequently asked questions about latent class analysis. Transl. Issues Psychol. Sci..

[30] Wurpts IC, Geiser C (2014). Is adding more indicators to a latent class analysis beneficial or detrimental? Results of a Monte-Carlo study. Front. Psychol..

[31] Weller BE, Bowen NK, Faubert SJ (2020). Latent class analysis: A guide to best practice. J. Black Psychol..

[32] Kim KM (2021). Prevalence, disability, and management patterns of migraine in Korea: Nationwide survey data from 2009 and 2018. J. Clin. Neurol..

[33] Korea & Statistics. *Korean Statistical Information Service, Statistical Database, Population, Households and Housing Unit*, http://kosis.kr/eng/statisticsList/statisticsListIndex.do?menuId=M_01_01&vwcd=MT_ETITLE&parmTabId=M_01_01&statId=1962001&themaId=#SelectStatsBoxDiv. (2018).

[34] Eriksen MK, Thomsen LL, Olesen J (2005). The visual aura rating scale (VARS) for migraine aura diagnosis. Cephalalgia.

[35] Kim BK, Cho S, Kim HY, Chu MK (2021). Validity and reliability of the self-administered visual aura rating scale questionnaire for migraine with aura diagnosis: A prospective clinic-based study. Headache.

[36] Stewart WF (2000). Validity of the migraine disability assessment (MIDAS) score in comparison to a diary-based measure in a population sample of migraine sufferers. Pain.

[37] Yang M, Rendas-Baum R, Varon SF, Kosinski M (2011). Validation of the headache impact test (HIT-6) across episodic and chronic migraine. Cephalalgia.

[38] Lee HS, Chung CS, Song HJ, Park HS (2000). The reliability and validity of the MIDAS (migraine disability assessment) questionnaire for Korean migraine sufferers. J. Korean Neurol. Assoc..

[39] Chu M-K (2009). Validity and reliability assessment of Korean headache impact test-6 (HIT-6). J. Korean Neurol. Assoc..

[40] Kroenke K, Spitzer RL, Williams JB, Monahan PO, Lowe B (2007). Anxiety disorders in primary care: Prevalence, impairment, comorbidity, and detection. Ann. Intern. Med..

[41] Ahn JK, Kim Y, Choi KH (2019). The psychometric properties and clinical utility of the Korean Version of GAD-7 and GAD-2. Front. Psychiatry.

[42] Kroenke K, Spitzer RL (2002). The PHQ-9: A new depression diagnostic and severity measure. Psychiatr. Ann..

[43] Seo JG, Park SP (2015). Validation of the generalized anxiety disorder-7 (GAD-7) and GAD-2 in patients with migraine. J. Headache Pain.

[44] Seo JG, Park SP (2015). Validation of the patient health questionnaire-9 (PHQ-9) and PHQ-2 in patients with migraine. J. Headache Pain.

[45] Lipton RB (2008). Cutaneous allodynia in the migraine population. Ann. Neurol..

[46] Krupp LB, LaRocca NG, Muir-Nash J, Steinberg AD (1989). The fatigue severity scale: Application to patients with multiple sclerosis and systemic lupus erythematosus. Arch. Neurol..

[47] Cho YW (2011). The reliability and validity of the Korean version of the Epworth sleepiness scale. Sleep Breath.

[48] Dean N, Raftery AE (2010). Latent class analysis variable selection. Ann. Inst. Stat. Math..

[49] Linzer DA, Lewis JB (2011). poLCA: An R package for polytomous variable latent class analysis. J. Stat. Softw..

[50] Dinno A (2015). Nonparametric pairwise multiple comparisons in independent groups using Dunn's test. Stata J..

[51] R Core team. *R: A Language and Environment for Statistical Computing v. 3.6.0* (R Foundation for Statistical Computing, 2019).

