# Supplementary material for: Classifying migraine subtypes and their characteristics by latent class analysis using data of a nation-wide population-based study
Source: Sci Rep. 2021 Nov 3;11:21595. doi: 10.1038/s41598-021-01107-7 (PMC8566532; doi:10.1038/s41598-021-01107-7)
Supplement: Supplementary file 1 — Supplementary Information. [file 41598_2021_1107_MOESM1_ESM.docx]

**Classifying migraine subtypes and their characteristics by latent class analysis using data of a nation-wide population-based study**

Wonwoo Lee, In Kyung Min, Kwang Ik Yang, Daeyoung Kim, Chang-Ho Yun, Min Kyung Chu

Supplementary Table S1. Sociodemographic distribution of the total Korean population, survey participants, and participants with migraine

|  | **Total population**  **(n=42394045)** | **Survey participants**  **(n=2501)** | **P-value** | **Participants with migraine**  **(n=125)** |
| --- | --- | --- | --- | --- |
| Age, years |  |  |  |  |
| 19–29 | 7624184 (17.8) | 434 (17.4) | 1.000 | 19 (15.2%) [8.9–21.5] |
| 30–39 | 7446677 (17.3) | 425 (17.0) |  | 33 (26.4%) [18.7–32.1] |
| 40–49 | 8408883 (19.6) | 498 (19.9) |  | 24 (19.2%) [12.3–26.1] |
| 50–59 | 8515725 (19.8) | 498 (19.9) |  | 23 (18.4%) [11.5–25.2] |
| 60–69 | 5854493 (13.6) | 345 (13.8) |  | 15 (12.0%) [6.3–17.7] |
| ≥70 | 5082994 (11.8) | 301 (12.0) |  | 11 (8.8%) [3.8–13.8] |
| Sex |  |  | 0.977 |  |
| Male | 21024909 (48.9) | 1242 (49.6) |  | 50 (40.0%) [31.4–48.6] |
| Female | 21544156 (50.1) | 1259 (50.4) |  | 75 (60.0%) [51.4–68.6] |
| Education |  |  | 0.607 |  |
| Middle school or lower | 5194888 (12.1) | 393 (15.7) |  | 16 (12.8%) [6.9–18.7] |
| High school | 17130249 (39.9) | 1063 (42.5) |  | 52 (41.6%) [33.0–50.2] |
| College or higher | 20607819 (48.0) | 1045 (41.8) |  | 57 (45.6%) [36.9–54.3] |

Data are presented as the mean±standard deviation, n (%), or [95% confidence interval].

Supplementary Table S2. Estimated prior probabilities of latent class membership

| **Latent Class 1 (n=65) Estimate (Standard error)** | **Latent Class 2 (n=42) Estimate (Standard error)** | **Latent Class 3 (n=18) Estimate (Standard error)** |
| --- | --- | --- |
| 0.542 (0.051) | 0.333 (0.043) | 0.126 (0.040) |

Supplementary Table S3. Estimated class-conditional response probabilities

|  | **Latent Class 1 (n=65) Estimate (Standard error)** | **Latent Class 2 (n=42) Estimate (Standard error)** | **Latent Class 3 (n=18) Estimate (Standard error)** |
| --- | --- | --- | --- |
| Monthly headache frequency: <2 | 0.6868 (0.0640) | 0.5240 (0.0857) | 0.4269 (0.2077) |
| Monthly headache frequency: ≥2, <8 | 0.2643 (0.0599) | 0.2630 (0.0777) | 0.2651 (0.1632) |
| Monthly headache frequency: ≥8, <15 | 0.0189 (0.0195) | 0.1415 (0.0635) | 0.3080 (0.1543) |
| Monthly headache frequency: ≥15 | 0.0300 (0.0237) | 0.0714 (0.0441) | 0.0000 (0.0000) |
| Headache intensity: Mild | 0.1922 (0.0569) | 0.1920 (0.0655) | 0.0000 (0.0000) |
| Headache intensity: Moderate | 0.5447 (0.0724) | 0.7379 (0.0713) | 0.6642 (0.1434) |
| Headache intensity: Severe | 0.2631 (0.0587) | 0.0700 (0.0453) | 0.3358 (0.1434) |
| Unilateral location: Unilateral | 0.9147 (0.0408) | 0.4502 (0.0915) | 0.4041 (0.2038) |
| Unilateral location: Bilateral | 0.0853 (0.0408) | 0.5498 (0.0915) | 0.5959 (0.2038) |
| Pulsating quality: Y | 0.7449 (0.0602) | 0.7843 (0.0719) | 0.9519 (0.0726) |
| Pulsating quality: N | 0.2551 (0.0602) | 0.2157 (0.0719) | 0.0481 (0.0726) |
| Aggravation by routine physical activity: Y | 0.1565 (0.0708) | 0.7863 (0.0889) | 1.0000 (0.0000) |
| Aggravation by routine physical activity: N | 0.8435 (0.0708) | 0.2137 (0.0889) | 0.0000 (0.0000) |
| Nausea: Y | 0.9699 (0.0219) | 0.7364 (0.0972) | 1.0000 (0.0000) |
| Nausea: N | 0.0301 (0.0219) | 0.2636 (0.0972) | 0.0000 (0.0000) |
| Vomiting: Y | 0.5040 (0.0733) | 0.4762 (0.0955) | 0.1316 (0.1127) |
| Vomiting: N | 0.4960 (0.0733) | 0.5238 (0.0955) | 0.8684 (0.1127) |
| Photophobia: Y | 0.0175 (0.0183) | 1.0000 (0.0000) | 0.1416 (0.1089) |
| Photophobia: N | 0.9825 (0.0183) | 0.0000 (0.0000) | 0.8584 (0.1089) |
| Phonophobia: Y | 0.3248 (0.0677) | 1.0000 (0.0000) | 0.1541 (0.1439) |
| Phonophobia: N | 0.6752 (0.0677) | 0.0000 (0.0000) | 0.8459 (0.1439) |

Y: yes, N: no

Supplementary Table S4. Results of multiple comparisons of clinical features among the three migraine classes

|  | **Class 1 vs. Class 2** | **Class 1 vs. Class 3** | **Class 2 vs. Class 3** |
| --- | --- | --- | --- |
| **Headache intensity**^‡^ | 0.111 | 0.317 | 0.024^†^ |
| **Unilateral location**^‡^ | <0.001^†^ | <0.001^†^ | >0.999 |
| **Pulsating quality**^‡^ | >0.999 | 0.308 | 0.767 |
| **Aggravation by routine physical activity**^‡^ | <0.001^†^ | <0.001^†^ | 0.140 |
| **Nausea**^‡^ | 0.001^†^ | >0.999 | 0.074 |
| **Vomiting**^‡^ | >0.999 | 0.005^†^ | 0.022^†^ |
| **Photophobia**^‡^ | <0.001^†^ | 0.351 | <0.001^†^ |
| **Phonophobia**^‡^ | <0.001^†^ | 0.179 | <0.001^†^ |
| **Monthly headache frequency** |  |  |  |
| *n*^*^ | 0.160 | 0.067 | >0.999 |
| By category^‡^ | 0.120 | 0.018^†^ | >0.999 |
| **Headache duration (min)** ^*^ | >0.999 | 0.030^†^ | 0.173 |
| **Osmophobia**^‡^ | <0.001^†^ | 0.367 | 0.137 |
| **Aura (VARS score ≥ 3)** ^‡^ | 0.005^†^ | >0.999 | 0.336 |
| **VARS score**^*^ | 0.008^†^ | >0.999 | 0.283 |
| **Anxiety (GAD-7 score ≥ 8)** ^‡^ | 0.032^†^ | 0.887 | >0.999 |
| **Fatigue (FSS score ≥ 4)** ^‡^ | 0.042^†^ | 0.302 | >0.999 |
| **FSS**^*^ | 0.018^†^ | 0.683 | >0.999 |

Due to familywise type 1 error >0.05 correction was needed and adjusted P-values were obtained by Bonferroni correction.

Statistical methods for multiple comparisons:^*^Dunn procedure for non-normally distributed variables or ^‡^Chi-square test or Fisher’s exact test for categorical variables.

^†^ Significant P-value

VARS: Visual Analogue Rating Scale, GAD-7: General Anxiety Disorder-7, FSS: Fatigue Severity Scale
